# Improvement in symptoms and pulmonary function of asthmatic patients due to their treatment according to the Global Strategy for Asthma Management (GINA)

**DOI:** 10.1186/1471-2466-8-26

**Published:** 2008-12-22

**Authors:** Mohammad H Boskabady, Fariba Rezaeitalab, Najmah Rahimi, Damon Dehnavi

**Affiliations:** 1 Department of Physiology, Ghaem Medical Centre, Mashhad University of Medical Sciences, Mashhad, Iran

## Abstract

**Background:**

Global Initiative Strategy for Asthma Management (GINA) is poorly applied in undeveloped and developing countries. The current study examined the effects of applying GINA guidelines on treatment efficacy in asthmatic patients in Iran.

**Methods:**

Twenty four asthmatic patients (usual care group) were treated as usual and 26 patients (intervention group) according to the GINA for 2 months. Asthma symptom score, asthma severity, frequency of symptoms/week and wheezing were recorded at the beginning (first visit), one month after treatment (second visit), and at the end of the study (third visit). Pulmonary function tests (PFTs) were performed by spirometry, and the patients' use of asthma drugs and their symptoms were evaluated, at each visit.

**Results:**

Asthma symptoms, frequency of symptoms/week, chest wheezing, and PFT values were significantly improved in the intervention group at the second and third visits compared to first visit (p < 0.001 for all measures). In addition, exercise induced cough and wheeze were significant improved in the third visit compared to the second visit in this group (p < 0.01 for both measures). In the second and third visits all symptoms were significantly lower, and PFT values higher, in the intervention group compared to the usual care group (p < 0.005 to p < 0.001). In the usual care group, there were only small improvements in some parameters in just the second visit (p < 0.01 for all measures). The use of asthma drugs was unchanged in the usual care group and significantly reduced in the intervention group (p < 0.01) by the end of the study.

**Conclusion:**

Adoption of GINA guidelines improves asthma symptoms and pulmonary function in asthmatic patients in Iran.

## Background

Asthma is known as a chronic disease, and epidemiological studies indicate that its prevalence is increasing worldwide [1]. The reason for the rise in prevalence is not clear [2]. Asthma affects over 10% of children and over 5% of adults in many European countries, and imposes a large burden on health services provision [1]. In Iran asthma affects 4.2% of children [3] and 2.8% of adults [4]. Optimal management of asthma requires appropriate drug administration, avoidance of exacerbating factors, and patient education [5].

There is still debate about how asthma should be treated pharmacologically and at what stage anti-inflammatory therapy should be introduced [6]. Low use of as-required beta2-agonist (less than once a day), and using drugs with low risk of side-effects are important goals of asthma treatment. However, sensitive indices of asthma severity including quality-of-life measurements, measurement of bronchial reactivity, or a measure of airway inflammation may be accessible to some types of health care services in developing countries. Lack of easily accessible guidelines, some medications and perhaps spirometry in developing countries making it difficult to assess asthma severity and achievement of optimal treatment. Thus asthma symptoms are often underestimated, leading to inadequate therapy [3].

The goals of asthma treatment have been significantly altered in the new guidelines [5]. More attention is paid to patient satisfaction placing greater emphasis on quality of life goals and on the partnership between the patient and provider rather than the provider simply telling the patient what to do [6]. We have previously shown that asthmatic patients in Iran benefit poorly from asthma education programs designed to teach them to avoid risk factors and to administer drugs correctly (specially with inhalers) [3,4]. Other forms of education (e.g., proper use of inhalers, avoidance of aggravating factors, regular PEF measurements) did lead to significant improvement in asthma management [7-9].

In the present study, we examined the effect of adopting the Global Initiative Strategy for Asthma Management (GINA) to treat asthma patients in Iran on asthma symptoms (including wheezing) and spirometric measures of pulmonary function.

## Methods

### Patients

Fifty asthmatic patients were recruited from the Asthma Clinic, Ghaem Medical Centre, Mashhad University of Medical Sciences and divided randomly into a usual care group (24 patients, 15 female, aged 45 ± 11 years, height 159 ± 8 cm) and a intervention group (26 patients,16 female, aged 40 ± 11 years, height 162 ± 9 cm). Inclusion criteria were: 1) previously diagnosed with asthma by a physician. 2) Two or more of the following symptoms: recurrent wheeze, cough or chest-tightness at rest; nocturnal or early morning wheeze, cough or chest-tightness; and wheeze or cough during exercise, 3) FEV1 and PEF less than 80% predicted values, 4) No history or symptoms of cardiovascular or other respiratory diseases that required treatment (excluding the common cold). The studied patients had moderate to severe asthma according to GINA guidelines [5]. The protocol was approved by the Ethics Committee of our institution, and each subject gave informed consent. The study was carried out during the Spring and Summer of 2005.

### Treatment duration and administered drugs

Each patient was treated for a period of two months and was visited and evaluated three times over this period. The treatment regimen of all patients included inhaled corticosteroid, mostly beclomethasone dipropionate (400–1400 μg depending on disease severity) and in some cases fluticasone dipropionate (500 μg). The treatment regimen included oral corticosteroids in 64% of the patients. Ninety-seven percent of patients received methyl xanthine and 58% received inhaled salbutamol. None of the patients had oral β-agonist drugs as part of their treatment regimen. Very few patients were under an acceptable therapeutic regimen for asthma at the start of the study according to GINA guidelines [5].

### Protocol

The intervention group was treated according to the GINA guidelines [5]. They were educated regarding the; 1) avoidance of risk factors for aggravation of the disease, 2) correct inhalation technique for using inhaler drugs, 3) regular usage of administration drugs. 3) recording of number and time of asthma attack. The treatment regimen of this group was adjusted and tapered according to symptoms score and PFT values. However, usual care group were treated by respirologist in the usual manner. The study was performed in single blind manner. Medical examination was performed and asthma symptoms taken at the beginning, middle (one month after starting the study) and end of the study for each patient. Asthma symptom score was counted according to Table 1[7,8,10]. The degree of wheezing was considered between 0 – 3 as follows: no wheezing = 0, hardly heard wheezing = 1, moderate wheezing = 2, and loud wheezing = 3.

**Table 1:** The criteria for asthma severity score

Symptom	Frequency	Score
Night wheezing	None	0
	Sleeping well with a little wheezing	1
	Waking once at night	2
	Waking most of night	3
Night cough	None	0
	Sleeping well with a little cough	1
	Waking once at night	2
	Waking most of night	3
Exercise cough and wheezing	No existence during strong exercise	0
	Existence only during strong exercise	1
	Existence during climbing stairs	2
	Existence during ordinary activity	3
Morning cough, tightness, and wheezing	None	0
	Existence in case of exertion	1
	Mild symptoms without exertion	2
	Waking at the morning due to symptoms	3
Day time cough, tightness, and wheezing	None	0
	Once a day	1
	Two or more times a day	2
	Affecting day time activity	3

Total score		16

Pulmonary function tests were also measured in the beginning and at the end of the study using a spirometer with a pneumotachograph sensor (Model ST90, Fukuda, Sangyo Co., Ltd. Japan). Prior to pulmonary function testing, the required manoeuvre was demonstrated by the operator, and subjects were encouraged and supervised throughout test performance. Pulmonary function testing was performed using the acceptability standards outlined by the American Thoracic Society (ATS) with subjects in a standing position and wearing nose clips [11]. All tests were carried out between 1000 and 1700 hours. Pulmonary function tests were performed three times in each subject. The highest level for forced vital capacity (FVC), forced expiratory volume in one second (FEV_1_), peak expiratory flow (PEF), maximal mid expiratory flow (MMEF), maximal expiratory flow at 75%, 50% and 25% of the FVC (MEF_75_, MEF_50 _and MEF_25 _respectively) were taken independently from the three curves.

### Data analysis

Based on the prevalence of asthma in Iran, using the PPS sampling method, it was calculated that a minimum of 15 subjects in each group would be needed to detect a 4% difference with an α error of 1% and a power of 95%. However, 24 patients as usual care and 26 patients as intervention group were studied. The data of asthma symptom score, chest wheeze, and frequency of occurrence of symptoms/week were expressed as mean± SEM and PFT values and those of height and age as mean± SD. All data were compared between the beginnings, middle and the end of the study (three visits) using repeated measure analysis of variance (ANOVA) and between usual care and intervention groups using unpaired "t" test. Due to the time-group interaction of the data, multivariate method (repeated measurements) was not applied for comparison of the beginnings, middle and the end of the study (three visits) between usual care and intervention groups. The difference of percentage of patients using each type of drug between each two visits was tested by Chi-square testing on 2 × 2 contingency tables. Significance was accepted at p < 0.05.

## Results

### Asthma symptoms

**Figure 1:**
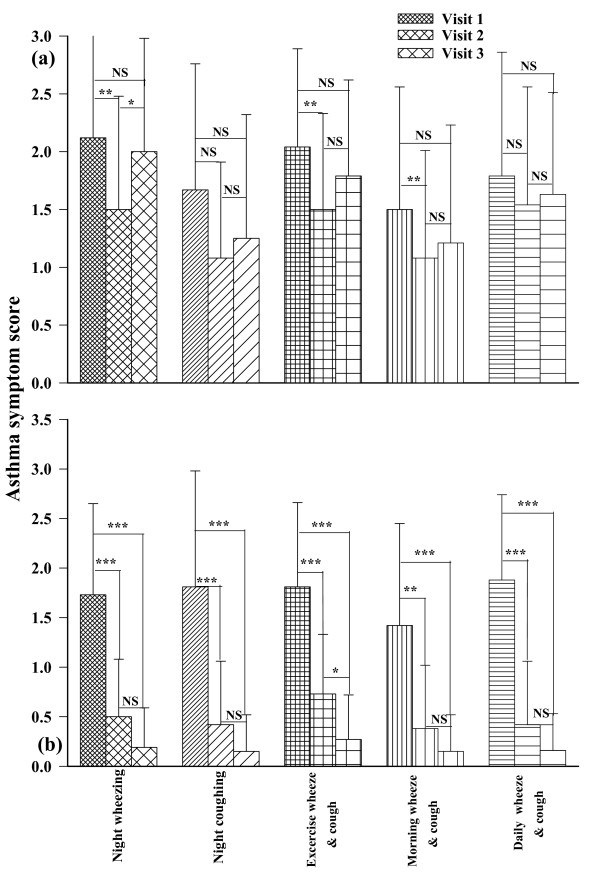
**Comparison of symptom score of usual care (a) and intervention (b) groups of asthmatic patients at the beginning (fine filled bars), middle (medium filled bars) and at the end (coarse filled bars) of a two month study period**. Statistical difference in different parameter between three visits: NS; non significant difference, *; p < 0.05, **; p < 0.01, ***, p < 0.001.

All symptom scores of asthmatic patients treated according to GINA guidelines (intervention group) were improved even after 1 month's treatment (second visit) and at the end of the study (third visit, (p < 0.001 for all cases). In addition, exercise induced cough was significantly improved in the third visit compared to the second visit in this group (p < 0.01), (Fig. 1b). However, in the usual care group there was a small improvement in some symptoms between the first and second visits only (p < 0.01 for all cases), (Fig. 1a). Asthma symptoms were not different between the intervention and usual care groups at the start of the study and were significantly reduced in the intervention group compared to the usual care subjects at the second and third visits (p < 0.005 to p < 0.001), (Table 2).

**Table 2:** Asthma symptoms and severity in usual care and intervention groups of patients at the beginning, middle and the end of the study

	Beginning		Middle		End	
			
Symptoms	Usual care Gr.	Intervention Gr.	Usual care Gr.	Intervention Gr.	Usual care Gr.	Intervention Gr.
Night wheezing	2.12 ± 0.90	1.73 ± 0.92 NS	1.50 ± 0.98	0.50 ± 0.58 ***	2.00 ± 0.98	0.19 ± 0.40 ***
Night coughing	1.67 ± 1.09	1.81 ± 1.17 NS	1.08 ± 0.83	0.42 ± 0.64 **	1.25 ± 1.07	0.15 ± 0.37 ***
Exercising W and C	2.04 ± 0.85	1.81 ± 0.85 NS	1.50 ± 0.83	0.73 ± 0.60 ***	1.79 ± 0.83	0.27 ± 0.45 ***
Morning W and C	1.50 ± 1.06	1.42 ± 1.03 NS	1.08 ± 093	0.38 ± 0.64 **	1.21 ± 1.02	015 ± 0.37 ***
Daily W and C	1.79 ± 1.02	1.88 ± 0.86 NS	1.54 ± 1.02	0.42 ± 0.64 ***	1.63 ± 0.88	0.16 ± 0.37 ***
Weekly W and C	5.79 ± 2.25	5.23 ± 1.90 NS	4.67 ± 1.97	1.38 ± 1.17 ***	5.46 ± 2.06	0.35 ± 0.56 ***
Chest wheezing	2.33 ± 0.76	2.19 ± 0.63 NS	1.83 ± 0.76	0.54 ± 0.65 ***	2.13 ± 0.80	0.31 ± 0.47 ***
Asthma severity	2.67 ± 1.01	2.23 ± 0.82 NS	2.38 ± 0.88	1.11 ± 0.52 ***	2.54 ± 1.02	1.00 ± 0.40 ***

Gr.: group, W: wheezing, C: coughing. All values were quoted as mean ± SEM. Statistical difference in different parameter between usual care and intervention group: NS; non significant difference, **; p < 0.005, ***; p < 0.001.

### Severity of asthma and wheezing

**Figure 2:**
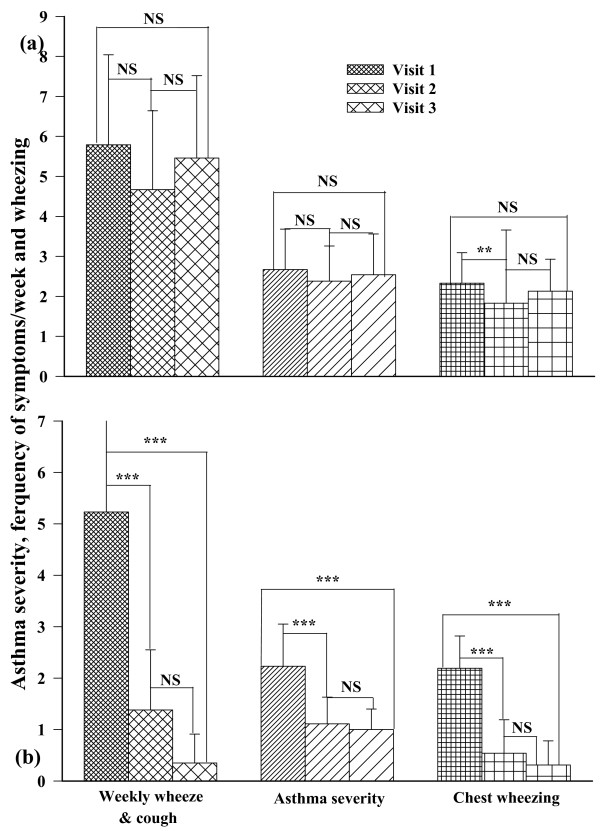
**Comparison of severity of asthma according to GINA guidelines, frequency of asthma symptoms/week and chest wheeze of usual care (a) and intervention (b) groups of asthmatic patients at the beginning (fine filled bars), middle (medium filled bars) and at the end (coarse filled bars) of a two month study**. Statistical difference in different parameter between three visits: NS; non significant difference, *; p < 0.05, **; p < 0.01, ***, p < 0.001.

Asthma severity score, frequency of occurrence of asthma symptoms/week and chest wheezing were also control improved at the second and third visit in the intervention group (p < 0.001 for each measure). The symptoms/week continued to control improve significantly between the second and third visits in this group (p < 0.01), (Fig. 2b). In the usual care group only chest wheezing was significantly control improved at the second visit than at the first visit (p < 0.05) but were not significantly difference between third and first visits (Fig. 2a). While at the beginning of the study, there was no significant difference in asthma severity score, frequency of asthma symptoms/week and chest wheezing between the usual care and intervention groups, in second and third visit all parameters in the intervention group became significantly lower than usual care group (p < 0.001 for all cases), (Table 2).

### Pulmonary function tests

**Figure 3:**
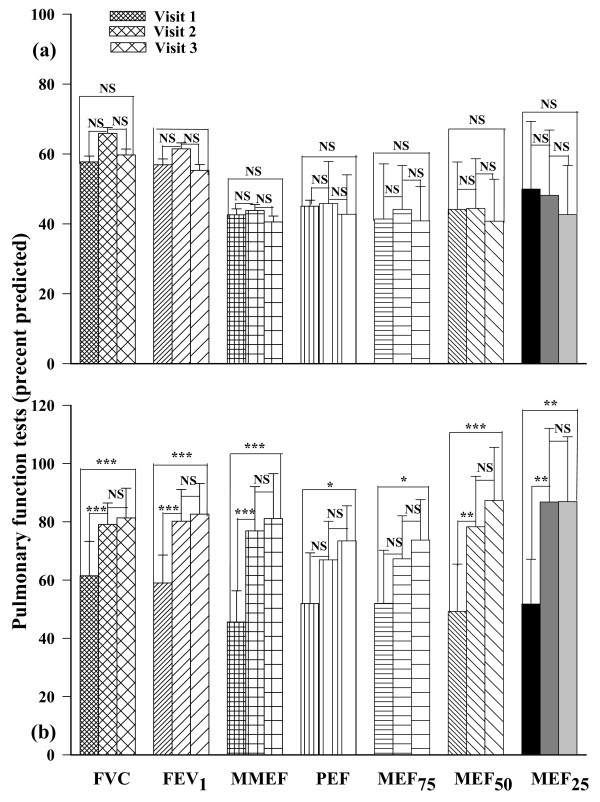
**Comparison of pulmonary function tests of usual care (a) and intervention (b) groups of asthmatic patients at the beginning (fine filled bars), middle (medium filled bars) and at the end (coarse filled bars) of a two month study**. FEV_1_: forced expiratory volume in one second; FVC: forced vital capacity; PEF: peak expiratory flow; MMEF: maximal mid expiratory flow; MEF_75_, MEF_50 _and MEF_25_: maximal expiratory flow at 75%, 50%, and 25% of the FVC respectively. All values of PFTs were quoted as percentage predicted. Statistical difference in different parameter between three visits: NS; non significant difference, *; p < 0.05, **; p < 0.01, ***, p < 0.001.

All PFT variables were abnormally low in both groups of patients at the beginning of the study (41.38 ± 15.77 to 61.50 ± 11.77 percent predicted values). In the intervention group, all PFT values were significantly higher in the second visit (p < 0.01) and the third visit (p < 0.001) compared to the first (Fig 3b). Since PFTs were not significantly different between the second and third visits in this group, the improvement occurred primarily within 1 month. In the usual care group, only FVC was significantly improved in the second visit compared to the first visit (p < 0.05), (Fig. 3a). While there was no significant difference in PFT variables between usual care and intervention groups at the beginning of the study, all PFT variables were significantly higher in the intervention group at the second and third visits (p < 0.001 for all measures), (Table 3).

**Table 3:** Pulmonary function tests (PFTs) in usual care and intervention groups of asthmatic patients at the beginning, middle and the end of the study

	Beginning		Middle		End	
			
PFTs	Usual care Gr.	Intervention Gr.	Usual care Gr.	Intervention Gr.	Usual care Gr.	Intervention Gr.
FVC	57.71 ± 10.57	61.50 ± 11.77 NS	65.88 ± 16.27	79.12 ± 7.35 ***	59.71 ± 10.10	81.35 ± 10.16 ***
FEV_1_(l)	56.88 ± 14.27	59.00 ± 9.57 NS	61.46 ± 16.23	80.19 ± 10.93 ***	55.29 ± 12.64	82.65 ± 10.50 ***
PEF(l/s)	45.04 ± 13.71	51.92 ± 17.43 NS	45.79 ± 12.05	66. 96 ± 13.21 ***	42.75 ± 11.28	73.46 ± 12.04 ***
MMEF	42.58 ± 17.04	45.61 ± 10.73 NS	43.83 ± 17.11	76.88 ± 15.18 ***	40.54 ± 14.05	81.12 ± 15.40 ***
MEF_75_(l/s)	41.38 ± 15.77	52.04 ± 18.21 *	44.17 ± 12.54	67.31 ± 14.75 ***	40.83 ± 9.85	73.73 ± 13.92 ***
MEF_50_(l/s)	44.13 ± 13.56	49.23 ± 16.23 NS	44.42 ± 14.21	78.31 ± 17.31 ***	40.75 ± 12.03	87.31 ± 18.26 ***
MEF_25_(l/s)	49.92 ± 19.40	51.77 ± 15.37 NS	48.17 ± 18.65	86.81 ± 25.28 ***	42.63 ± 14.08	86.96 ± 22.23 ***

Gr.: group, FEV_1_: forced expiratory volume in one second; FVC: forced vital capacity; PEF: peak expiratory flow; MMEF: maximal mid expiratory flow; MEF_75_, MEF_50 _and MEF_25_: maximal expiratory flow at 75%, 50%, and 25% of the FVC respectively. All values of PFTs were quoted as mean ± SD of percentage predicted. Statistical difference in different parameter between usual care and intervention groups: NS; non significant difference, *; p < 0.05, **; p < 0.005, ***; p < 0.001.

### Treatment regimen and inhaler using technique

The use of inhaled and oral β-agonists, inhaled and oral corticosteroid, and oral theophylline was decreased at the end of the study for the intervention group; the proportion of patients employing the correct technique when using their inhaler increased from 31% (visit 1) to 100% (visit 3) in this group. No obvious changes noted for the usual care group with regard to drug use or proportion of patients employing the correct technique when using their inhaler (table 4). In Table 5, number and percentage of two groups of asthmatic subjects under combination therapy and different type of drugs used and percentage of patients using each type of drug was presented.

**Table 4:** Different type of drugs in treatment regimen in usual care and intervention groups of asthmatic patients at the beginning, middle and the end of the study

Type of	Beginning		Middle		End	
			
Drugs	Usual care Gr.	Intervention Gr.	Usual care Gr.	Intervention Gr.	Usual care Gr.	Intervention Gr.
Inhaler salbotamol	75.00	66.70	83.00 NS	92.50 ***	79.20 NS ns	73.10 NS +++
Oral salbotamol	29.2	26.90	16.65 NS	15.40 *	25.00 NS ns	3.80 *** ++
Inhaler Salmetrol	0	3.80	20.8 ***	19.20 ***	4.2 NS +++	15.40 ** ns
Inhaler corticosteroid	25.00	26.90	62.50 ***	88.50 ***	41.7 * ++	30.80 NS +++
Oral corticosteroid	25.00	26.90	29.20 NS	42.30 *	20.80 NS ns	7.70 *** +++
Oral theophylline	50.00	53.85	83.00 ***	76.90 *	54.20 NS +++	15.40 *** +++
Anti-histamine	8.30	15.40	33.3 ***	30.80 *	8.30 NS +++	19.20 NS +
Inhaler using technique	41.70	30.80	45.80 NS	100 ***	45.80 NS ns	100 *** ns

(percentage of total patients in each group)

Statistical difference in the percentage of patients using each type of drug between beginning with middle and the end of the study: NS; non significant difference, *; p < 0.05, **; p < 0.005, ***; p < 0.001. Statistical difference in the percentage of patients using each type of drug between middle and the end of the study: ns; non significant difference, +; p < 0.05, ++; p < 0.005, +++; p < 0.001. The dose of beclomethasone dipropionate in patients of both groups was 400–1400 μg depending upon the severity of the disease and the dose of fluticasone dipropionate was 500 μg.

**Table 5:** Number and percentage of two groups of asthmatic subjects under combination therapy and different type of drugs used and percentage of patients using each type of drug

Type of Drugs	Usual care Gr.				Intervention Gr.			
	Beginning		End		Beginning		End	
	n	%	n	%	n	%	n	%
OBA+OCS+OT+IBA	6	25.0	5	20.8	7	27.0	1	3.8

OCS+OT+IBA	0	0.0	0	0.0	0	0.0	1	3.8
OBA+ OT+IBA +ICS	1	4.2	1	4.2	0	0.0	0	0.0
OT+IBA	0	0.0	5	20.8	5	19.2	0	0.0
OT+IBA+ ICS	5	20.9	2	8.3	2	7.7	2	7.7
LAIBA+ICS	0	0.0	1	4.2	1	3.8	4	15.4
IBA+AH	2	8,3	2	8.3	0	0.0	3	11.5
IBA+ ICS	0	0.0	2	8.3	0	0.0	1	3.8
AH+ICS	0	0.0	0	0.0	4	15.4	2	7.7
ICS only	0	0.0	2	8.3	0	0.0	1	3.8
IBA only	4	16.7	2	8.3	3	11.5	11	42.0

n: number, %: percentage of patients using each type of drug, OBA; oral beta agonist, OCS; oral corticosteroid, OT; oral theophylline, IBA; inhaled beta agonist, ICS; inhaled corticosteroid, LAIB; long acting inhaled beta agonist, AH; anti histamine

## Discussion

The results of the present study showed greater improvement in asthma symptoms and in pulmonary function of patients treated according to GINA guidelines comparing to a usual care group receiving usual treatment.

According to the guidelines the main aims of asthma management are to control symptoms maintain pulmonary function close to a normal level and maintain normal physical activity levels [5]. In the present study significantly greater improvements in PFT values were noted for the intervention group after only a short period of treatment according to GINA guidelines; the PFT values improved by more than 15% becoming close to predicted normal values in just 2 months oeriod. The asthma symptom scores also improved more in the intervention group, these patients were almost symptom free at the end of the study. Asthma severity improved from a moderate-severe persistent category to an intermittent-mild persistent category according to GINA guidelines. The chest wheeze of patients in studied group significantly reduced after two month's treatment. The amount and types of drugs in the treatment regimen of the intervention patients were decreased as a result of improvement in asthma severity. All patients in this group were better educated about their asthma, were able to use their inhaler correctly and were able to achieve almost normal daily activity levels by the end of the study. In contrast, only minimal changes were observed in symptom score and chest wheeze PFT values in the usual care group.

At the start of the study, the PFT values, symptoms score, asthma severity and chest wheeze tended to be worse in the usual care group compared to the intervention group, though these differences did not achieve statistical significance. This tendency may have reflected a slightly more severe level of disease and a greater expectation of treatment benefit in the usual care group. Thus, the short term improvement noted in the usual care group at the second visit, which were smaller in magnitude to that seen in the intervention group and were reversed by the third visit, were probably a placebo effect

The score of asthma symptoms used in the present study was based on questionnaires used in our previous studies (References 3, 4, 7, 8, 9 and 16). The preparation of the questionnaire was described in our previous study (Reference 4). Briefly, a questionnaire in the Farsi language was designed in accordance with several previous questionnaires and based on the definition of asthma used in the *Global Initiative for Asthma*.

Several previous studies have demonstrated the efficacy of applying various guidelines in the management of asthma. For example it has been shown that the regular measurement of PEF can lead to reduction of drug use in asthmatic patients, specially of bronchodilators and corticosteroids, [12,13], reduction of asthma symptoms and increase in PFT values [14], and better prediction of asthma attack [15]. It has also been suggested that regular PEF measurement is useful for determining the drug regimen in asthmatic patients [15]. Taken together, the above studies emphasize the benefit of employing standard guidelines in management of asthma.

However, in undeveloped and developing countries guidelines are poorly applied. Our previous studies showed that most asthmatic patients in Iran were not well treated and were not able to administer their inhaler drugs correctly [3,4,7,9,16]. Dashash and Mukhtar [17] also showed that in Saudi Arabia prescribing for asthmatic children did not conform to national guidelines for treatment of asthma. They recommend increasing the use of current asthma management guidelines by practitioners. In another study Hijazi et al showed that patient management is deficit in Kuwait, documentation was inadequate and adherence to the international guidelines was partial [18]. Other studies also suggest that major efforts should be directed to applying guidelines in the management of asthma [19].

Few studies have previously tested the effect of applying guidelines in the management of asthma in countries of our region. Alamoudi [20] examined the efficacy of a management protocol in reducing emergency visits and hospitalizations in chronic asthmatics. A reduction in emergency visits and hospitalizations of chronic asthmatics was demonstrated, irrespective of severity, by employing a simple protocol consisting of corticosteroids inhalation as a monotherapy and correction of the inhalation technique. Our previous studies also showed that regular PEF measurement and using rescue salbutamol inhaler at the time of chest tightness leads to improvement in FEV1 and PEF, reduction in symptom score, asthma attack and in rescue inhaled salbutamol use [7].

In addition to this, we have previously shown that most asthmatic patients in Iran did not receive adequate knowledge regarding avoidance of risk factors and of acceptable treatment regimens. The addition of a standard treatment plan in asthmatic patients led to improvement in symptom score and PFT values [8]. We have also previously emphasised that education is an important and necessary step in the efficacy of inhaler drugs. Most asthmatic patients did not receive adequate instruction regarding the inhalation technique and were not able to use their inhaler correctly; once fully educated in this regard, salbutamol inhalation by the inhaler alone was as effective as using it with a spacer. [9].

The application of GINA guideline 2002 in treating asthmatic patients showed significant improvement in asthma therapy. Therefore, the application of GINA guideline 2006 my resulted in greater improvement in treating asthmatic patients. In addition, in the present study patients were studied for relatively short period of time (2 months) and treating patients for longer period could lead to better results. However in this study period, there was significant improvement in treatment of patients. This may be due to education and style deficiency in treating asthmatic patient in our country.

Although the questionnaire used in the present study was validated in the previous studies [7,8,10], there are some limitation in this study including using version 2002 of GINA guidelines in the study and therefore the data are not new. The treatment received by the participants was also a bit heterogeneous which is the nature of treatment regimen of the patients in developing countries. The QoL should be 'evaluated by an international validated questionnaire. However, the results of the study are interesting and therefore further studies need to be performed according new version of GINA guideline and using international validated questionnaire in devolving countries.

## Conclusion

The present study extends the findings of our previous studies by demonstrating the suitability of adopting GINA guidelines in the management of asthma in countries of our region. The demonstration of significant improvements in asthma symptoms (specially chest wheeze), severity, drug usage, inhalation technique, and PFT values after applying GINA guidelines is promising. However, further studies are needed with greater numbers of patients with a wider range of disease severity. The long term benefits will also need to be tested by monitoring the effects over a longer period.

## Pre-publication history

The pre-publication history for this paper can be accessed here:


